# Integrative annotation and knowledge discovery of kinase post-translational modifications and cancer-associated mutations through federated protein ontologies and resources

**DOI:** 10.1038/s41598-018-24457-1

**Published:** 2018-04-25

**Authors:** Liang-Chin Huang, Karen E. Ross, Timothy R. Baffi, Harold Drabkin, Krzysztof J. Kochut, Zheng Ruan, Peter D’Eustachio, Daniel McSkimming, Cecilia Arighi, Chuming Chen, Darren A. Natale, Cynthia Smith, Pascale Gaudet, Alexandra C. Newton, Cathy Wu, Natarajan Kannan

**Affiliations:** 1 Institute of Bioinformatics, University of Georgia, Athens, GA 30602 USA; 2 Protein Information Resource (PIR), Department of Biochemistry and Molecular & Cellular Biology, Georgetown University Medical Center, Washington, DC 20007 USA; 3 Department of Pharmacology, University of California, San Diego, La Jolla, CA 92093 USA; 4 The Jackson Laboratory, Bar Harbor, ME 04609 USA; 5 Department of Computer Science, University of Georgia, Athens, GA 30602 USA; 6 Department of Biochemistry & Molecular Pharmacology, NYU School of Medicine, New York, NY 10016 USA; 7 Genome, Environment, and Microbiome (GEM) Center of Excellence, University at Buffalo, Buffalo, NY 14203 USA; 8 Center for Bioinformatics and Computational Biology, University of Delaware, Newark, DE 19711 USA; 9 SIB Swiss Institute of Bioinformatics, Lausanne, 1015 Switzerland

## Abstract

Many bioinformatics resources with unique perspectives on the protein landscape are currently available. However, generating new knowledge from these resources requires interoperable workflows that support cross-resource queries. In this study, we employ federated queries linking information from the Protein Kinase Ontology, iPTMnet, Protein Ontology, neXtProt, and the Mouse Genome Informatics to identify key knowledge gaps in the functional coverage of the human kinome and prioritize understudied kinases, cancer variants and post-translational modifications (PTMs) for functional studies. We identify 32 functional domains enriched in cancer variants and PTMs and generate mechanistic hypotheses on overlapping variant and PTM sites by aggregating information at the residue, protein, pathway and species level from these resources. We experimentally test the hypothesis that S768 phosphorylation in the C-helix of EGFR is inhibitory by showing that oncogenic variants altering S768 phosphorylation increase basal EGFR activity. In contrast, oncogenic variants altering conserved phosphorylation sites in the ‘hydrophobic motif’ of PKCβII (S660F and S660C) are loss-of-function in that they reduce kinase activity and enhance membrane translocation. Our studies provide a framework for integrative, consistent, and reproducible annotation of the cancer kinomes.

## Introduction

Phosphorylation of serine, threonine and tyrosine residues by protein kinases is a major post-translational modification (PTM) that controls diverse regulatory and signalling mechanisms in cells. Aberrant phosphorylation is emerging as a common pathologic mechanism, and protein kinases are significantly overrepresented among mutant proteins in cancers^1^. It is even thought that in most tumours, at least one protein kinase or phosphatase gene is affected by mutation, copy number variation, or genetic rearrangements^2^. There is high interest in protein kinases as drug targets, and a growing number of protein kinase inhibitors are gaining FDA approval for therapeutic use, especially in cancer^3^. For example, mutations in the EGFR protein kinase domain are found in 10–30% of lung adenocarcinomas, and treatment with tyrosine kinase inhibitors that specifically target the mutant protein kinase can produce temporary remissions in lung cancer patients^4^.

Protein kinase activity is often itself controlled by PTMs, with phosphorylation of activation loop residues increasing the activity of most protein kinases^5^. The classic example of this mechanism is receptor tyrosine kinase (RTK) signalling, in which a ligand binding to its target receptor leads to dimerization, trans-autophosphorylation, and full kinase activation^6^. Thus, mutations that disrupt PTM of protein kinases could be potentially oncogenic.

Advances in next-generation sequencing and mass spectrometry proteomics technologies have led to an explosion of data on PTM sites and disease-associated genetic variations. Detection of sites and variants has far outpaced functional characterization and annotation of the corresponding data. Thus, for the vast majority of PTM sites and cancer-associated mutations, there is no annotation indicating how or even whether protein function is affected. In addition, many protein kinases are poorly studied, further hindering the annotation of PTMs and disease variants.

In order to gain insight into the role of PTMs in cancer, it is increasingly necessary to bring together data from diverse sources. There are numerous gene/protein bioinformatics resources that curate a variety of non-overlapping information, including PTMs and disease mutations. In addition to the comprehensive protein resource, UniProt^7^, there are resources that specialize in specific species such as neXtProt^8^ for human and the Mouse Genome Informatics^9^ (MGI) for mouse. The Protein Ontology^10^ (PRO) and iPTMnet^11^ capture specific data types on proteoforms and post-translational modifications (PTMs), respectively, across multiple species. The Catalog of Somatic Mutations in Cancer^12^ (COSMIC) captures information on human cancer-associated variants and the Protein Kinase Ontology^13^ (ProKinO) captures information on the sequence, structure and functional aspects of protein kinase across species. Integrating information from these resources, each of which has its own unique focus, has the greatest potential for new discovery. In particular, integrating detailed structural and functional knowledge from domain-specific resources with curated information captured in large protein ontologies and data stores has the potential to provide high-resolution annotation of mutations and PTMs. Several resources (for example UniProtKB, PRO, neXtProt, and ProKinO) provide Resource Description Framework (RDF) triple store representations, which makes their content accessible for querying via “SPARQL Protocol and RDF Query Language” (SPARQL) endpoints, thereby facilitating data integration.

To generate an overall picture of our knowledge of protein kinases, we developed an annotation score that reflects the aggregated information available about each protein kinase from several protein bioinformatics resources. By comparing this score with a National Institutes of Health (NIH) metric based on protein kinase mentions in scientific articles and grant applications^14^, we identified protein kinases that are potentially under-curated (low annotation scores relative to literature/grant mentions) and protein kinases that are surprisingly richly annotated given relatively modest literature/grant mentions. Next, we identified 32 domains, including 19 protein kinase domains, in 27 protein kinases that are statistically enriched in both cancer-associated mutations and PTM sites. Within the enriched domains, there were 103 sites that are both post-translationally modified and mutated in cancer samples (“mutation-PTM overlapping sites”). To test the idea that PTMs in these domains are important for regulating kinase activity, we performed two case studies: one focusing on the protein kinase domain of EGFR, a highly studied and richly annotated protein kinase that has frequent gain-of-function mutations in cancer and the other focusing on the C-terminal domains of two members of the PKC family, PRKCB (PKCβ) and PRKCQ (PKCθ), which have loss-of-function mutations in cancer. For the EGFR study, we performed experiments and structural modelling to test the hypothesis that S768 phosphorylation is inhibitory and that oncogenic mutations at this position, especially S768I, increase catalytic activity. For the PKC study, we performed experiments to test the hypothesis that cancer-associated PKC hydrophobic motif (HF-motif) mutations are, in contrast, inhibitory to kinase function. This work demonstrates the power of aggregated federated queries to provide annotation at multiple levels of granularity that enables hypothesis generation that is not possible using individual data sources.

## Results

### Coverage of the kinome in protein bioinformatics resources

**Figure 1:**
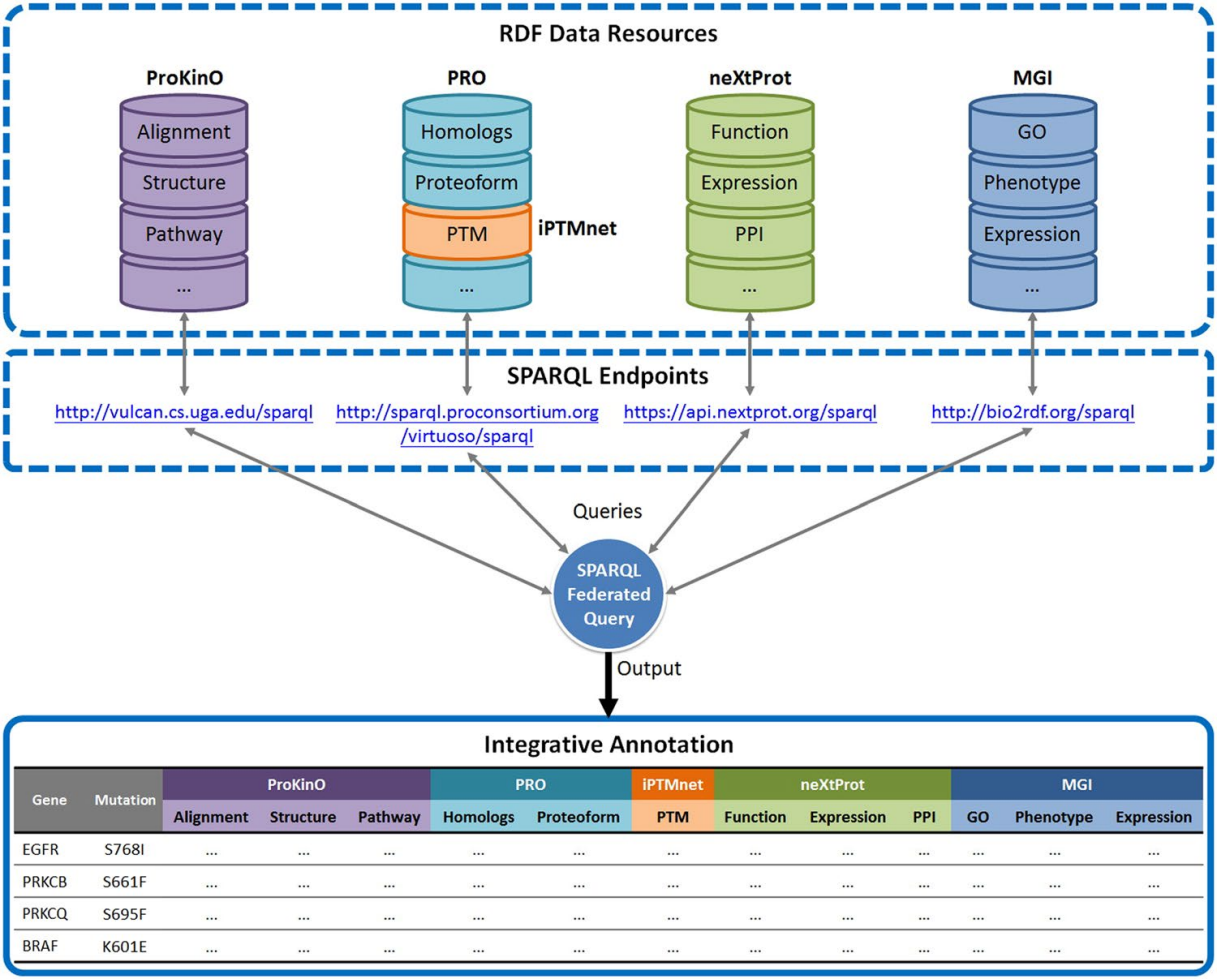
A framework for aggregate queries and integrative annotation. Integrative annotation across five resources, including ProKinO, PRO, iPTMnet, neXtProt, and MGI, is built using SPARQL federated query against four SPARQL endpoints; iPTMnet data is available via querying through PRO. Types of information are shown in each RDF data resource. PTM: post-translational modification; PPI: protein-protein interaction; GO: Gene Ontology.

Although protein kinases have been extensively studied for their role in human disease, much of our current understanding of kinase functions is skewed towards a subset of well-studied protein kinases. Assessing our level of knowledge about protein kinases is important because it allows us to identify knowledge gaps and to define future priorities for experimental and bioinformatics work. The National Institutes of Health (NIH) recently classified 134 protein kinases as “under-studied” or “dark kinases” using a metric based on the number of publications and the presence/absence of R01 funding: Jensen PubMed score^15^ <50, no R01s, and PubTator score^16^ <150. As a complement to the NIH literature-based score, we developed an annotation scoring metric, *ω*, based on the amount of information available in curated resources. We gathered information for all the human protein kinases from ProKinO, PRO, neXtProt, and iPTMnet and their mouse orthologs from MGI (Fig. 1) using a high-level SPARQL federated query (see Methods and Supplementary Fig. S1a) for all resources except iPTMnet, which does not currently have an RDF representation and calculated the score as described in Methods. A heatmap of the results shows that *ω* ranged from 7.82 for the best-annotated protein kinase, EGFR, to 0.22 for the least well-annotated protein kinase, PSKH2 (Supplementary Data S1). Nearly 200 protein kinases (~35% of the human kinome) had scores of less than 1, which we define as low-annotated protein kinases.

**Figure 2:**
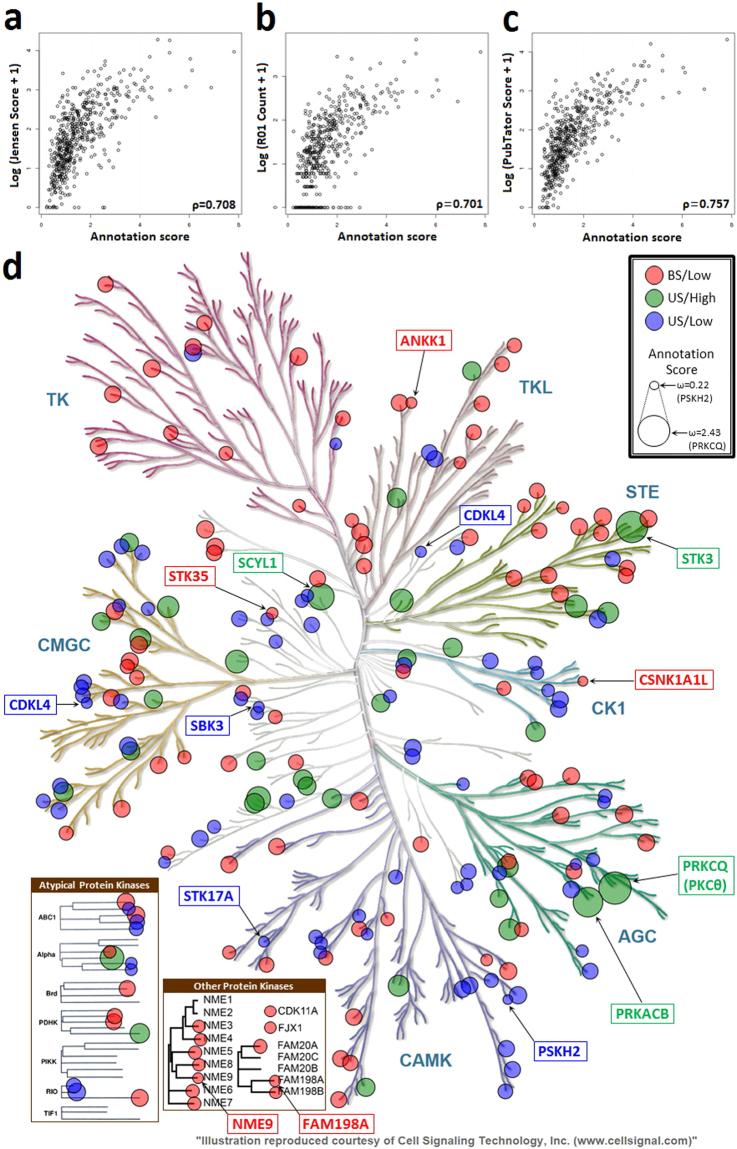
NIH metric versus annotation score. Scatter plots of annotation score *ω* versus the three measurements (in log scale) used in the NIH metric: (**a**) Jensen score, (**b**) R01 count, and (**c**) PubTator score, respectively. Spearman’s rank correlation coefficient *ρ* is shown in each scatter plot. (**d**) Visualization of the three annotation categories in the human kinome tree. BS/Low: better-studied protein kinases with low *ω* (red nodes); US/High: under-studied protein kinases with high *ω* (green nodes); US/Low: under-studied protein kinases with low *ω* (blue nodes); all the unmarked protein kinases are better-studied protein kinases with high *ω*, such as EGFR. Node size scales with the *ω* score. The five proteins with the highest *ω* in US/High category are labelled; the five proteins with the lowest *ω* in BS/Low and US/Low categories, respectively, are also labelled. The human kinome tree was generated using KinMap^43^. Illustration is reproduced courtesy of Cell Signaling Technology, Inc. (http://www.cellsignal.comwww.cellsignal.com).

Next, we compared our annotation score *ω* to the NIH metric (Fig. 2 and Supplementary Data S1). Comparisons between *ω* and the three measurements used in NIH metric, Jensen PubMed score, R01 count, and PubTator score, are presented in Fig. 2a to c, respectively. Although the figures show high Spearman correlations between *ω* and the three measurements (Spearman’s rank correlation coefficient *ρ* = 0.708, *ρ* = 0.701, and *ρ* = 0.757, respectively), the literature-based scores do not entirely reflect the degree to which protein kinases are annotated in bioinformatics resources. Based on the criteria for the three measurements defined by the NIH metric, we found that 316 protein kinases (59% of the human kinome) were considered better-studied (BS) by the NIH metric and had *ω* greater than 1 (BS/High). These protein kinases are those that are being actively studied experimentally and are richly annotated in bioinformatics resources. On the other hand, there were 80 protein kinases (15% of the human kinome) that were considered under-studied (US) by the NIH metric and had *ω* less than 1 (US/Low). These are protein kinases that need additional experimental characterization with the subsequent curation of the results. US/Low protein kinases are mainly distributed among Ser/Thr protein kinase families (Fig. 2d, blue nodes), including the CAMK, CMCG, and ACG groups (19%, 17.5%, and 15% of the US/Low category, respectively); US/Low protein kinases are less common in the tyrosine kinase (TK), tyrosine kinase-like (TKL), and STE groups (all are 2.5% of the US/Low category).

For the remaining protein kinases, the classifications by the NIH metric and our annotation score *ω* are not aligned. The two methods are measuring different aspects of protein kinase knowledge–the NIH metric is measuring unstructured knowledge (mentions in the literature) and research interest (mentions in grant applications) whereas *ω* is measuring curated information–so these protein kinases are potentially interesting cases. First, there are 108 protein kinases (20% of the human kinome) that are considered better-studied by the NIH metric but received *ω* less than 1 (BS/Low). This suggests that there is information available about these protein kinases that are not represented in curated resources or that these protein kinases are currently of high interest (i.e., are being mentioned in grant application), which could lead to newly published data in the near future. In either case, these protein kinases would be good candidates to prioritize for further curation. Although BS/Low protein kinase are found across the kinome (Fig. 2d, red nodes), the proportion of CAMK and STE groups in this category is particularly high (13% and 12% of the BS/Low category, respectively), indicating that special attention should be paid to these groups.

Finally, there were 34 protein kinases (6% of the human kinome) that were considered under-studied by the NIH metric but had *ω* greater than 1 (US/High). There are multiple reasons why US/High protein kinases may be more annotation-rich than their score by the NIH metric suggests. First, they may be annotated with data from high-throughput genomic or proteomic experiments. Results of these experiments are often reported only in supplemental data tables or are submitted directly to bioinformatics resources and would be missed by the NIH metric, which counts mentions in scientific articles. Also, our annotation score *ω* includes information from MGI on the mouse orthologs of the human protein kinases. Results from model organisms can be very useful in augmenting the annotation of human proteins and may not be reflected in the NIH metric. The US/High protein kinases with highest *ω* belong to the ACG (PRKCQ and PRKACB), STE (STK3), and other (SCYL1 and NEK6) kinase groups (Fig. 2d, green nodes). Overall, US/High protein kinases are mainly in the CMGC group (23.5% of the US/High category); on the other hand, there are no US/High protein kinases in the TK group.

### Prioritization of overlapping PTMs and disease variants for annotation

There are 217,925 non-redundant protein kinase missense mutations across all cancer samples in COSMIC v81 (92,222 missense mutations in genome-wide screened samples) and 16,545 non-redundant PTMs (from iPTMnet) identified in the human kinome. Many of these mutations and PTMs map to critical functional domains such as the protein kinase domain as well as to regulatory domains. To prioritize key mutations and PTMs for functional studies and mechanistic annotation, we performed Variant Enrichment Analysis (VEA) and Modification Enrichment Analysis (MEA) (see Methods). VEA identifies functional domains with the higher-than-expected frequency of mutations across different cancer samples, while MEA identifies functional domains with the higher-than-expected number of known modified sites. We identified 32 domains in 27 protein kinases (Table 1) that are significantly enriched in both cancer-associated variants and PTMs. Protein kinase domains (19/32 enriched domains) were the most prevalent. Regulatory domains, including the C-terminal domains (Pkinase_C) of PRKCB (PKCβ) and PRKCQ (PKCθ), the Pleckstrin homology (PH) domain of AKT1, the P21-Rho-binding domain (PBD) of PAK2 and the transmembrane domain of EphA2 kinase (EphA2_TM), were also identified. Within the enriched domains, there are 103 positions that are both post-translationally modified and mutated in cancer samples (“mutation-PTM overlapping sites”; Supplementary Table S1).

**Table 1:** Domains enriched in both disease variants and PTMs. Adj-PV: adjusted p-value; Average score: an average of VEA score and MEA score; bold text: protein kinase domains (either Pkinase or Pkinase_Tyr).

Gene Symbol	UniProtKB ID	Domain	VEA Adj-PV	MEA Adj-PV	Average Score
**EGFR**	**P00533**	**Pkinase_Tyr**	**<1.00E-14**	**<1.00E-14**	**14.000**
**ERBB2**	**P04626**	**Pkinase_Tyr**	**4.32E-14**	**6.70E-06**	**9.269**
**BRAF**	**P15056**	**Pkinase_Tyr**	**<1.00E-14**	**3.42E-05**	**9.233**
**MAP2K3**	**P46734**	**Pkinase**	**<1.00E-14**	**5.33E-05**	**9.137**
**PRKCB**	**P05771**	**Pkinase**	**<1.00E-14**	**3.68E-04**	**8.717**
**CHEK2**	**O96017**	**Pkinase**	**<1.00E-14**	**6.68E-04**	**8.588**
**EPHA5**	**P54756**	**Pkinase_Tyr**	**7.55E-14**	**2.22E-03**	**7.888**
**FLT3**	**P36888**	**Pkinase_Tyr**	**1.84E-12**	**6.90E-04**	**7.448**
EPHA7	**Q15375**	**Pkinase_Tyr**	**1.53E-11**	**1.33E-04**	**7.345**
ZAP70	**P43403**	**Pkinase_Tyr**	**1.20E-03**	**4.61E-12**	**7.129**
**PRKACA**	**P17612**	**Pkinase**	**2.93E-09**	**8.13E-06**	**6.811**
**KIT**	**P10721**	**Pkinase_Tyr**	**6.81E-10**	**4.21E-04**	**6.271**
**LCK**	**P06239**	**Pkinase_Tyr**	**4.20E-03**	**3.39E-10**	**5.923**
**MAP2K1**	**Q02750**	**Pkinase**	**1.45E-04**	**1.26E-08**	**5.870**
**RET**	**P07949**	**Pkinase_Tyr**	**7.21E-04**	**1.82E-05**	**3.941**
**AXL**	**P30530**	**Pkinase_Tyr**	**6.76E-04**	**7.84E-05**	**3.638**
**CSNK1A1L**	**Q8N752**	**Pkinase**	**1.03E-04**	**2.01E-03**	**3.341**
**HCK**	**P08631**	**Pkinase_Tyr**	**5.29E-05**	**4.97E-03**	**3.290**
**FGFR2**	**P21802**	**Pkinase_Tyr**	**5.20E-04**	**3.47E-03**	**2.872**
AKT1	P31749	PH	<1.00E-14	2.12E-04	8.837
EGFR	P00533	GF_recep_IV	<1.00E-14	1.68E-03	8.387
PAK2	Q13177	PBD	2.15E-11	1.31E-03	6.775
PRKCB	P05771	Pkinase_C	3.68E-08	6.94E-05	5.797
EGFR	P00533	Recep_L_domain	6.07E-07	3.74E-04	4.822
EPHB2	P29323	EphA2_TM	4.08E-03	6.38E-07	4.293
EPHA3	P29320	EphA2_TM	5.75E-06	9.32E-04	4.136
TTN	Q8WZ42	PPAK	1.93E-04	4.29E-05	4.041
EPHB1	P54762	EphA2_TM	3.75E-04	1.60E-04	3.612
BRDT	Q58F21	Bromodomain	3.72E-04	1.66E-03	3.104
TTN	Q8WZ42	fn3	7.85E-04	9.89E-04	3.055
EPHA7	Q15375	EphA2_TM	2.05E-03	5.65E-04	2.968
PRKCQ	Q04759	Pkinase_C	3.83E-03	1.08E-03	2.691

**Figure 3:**
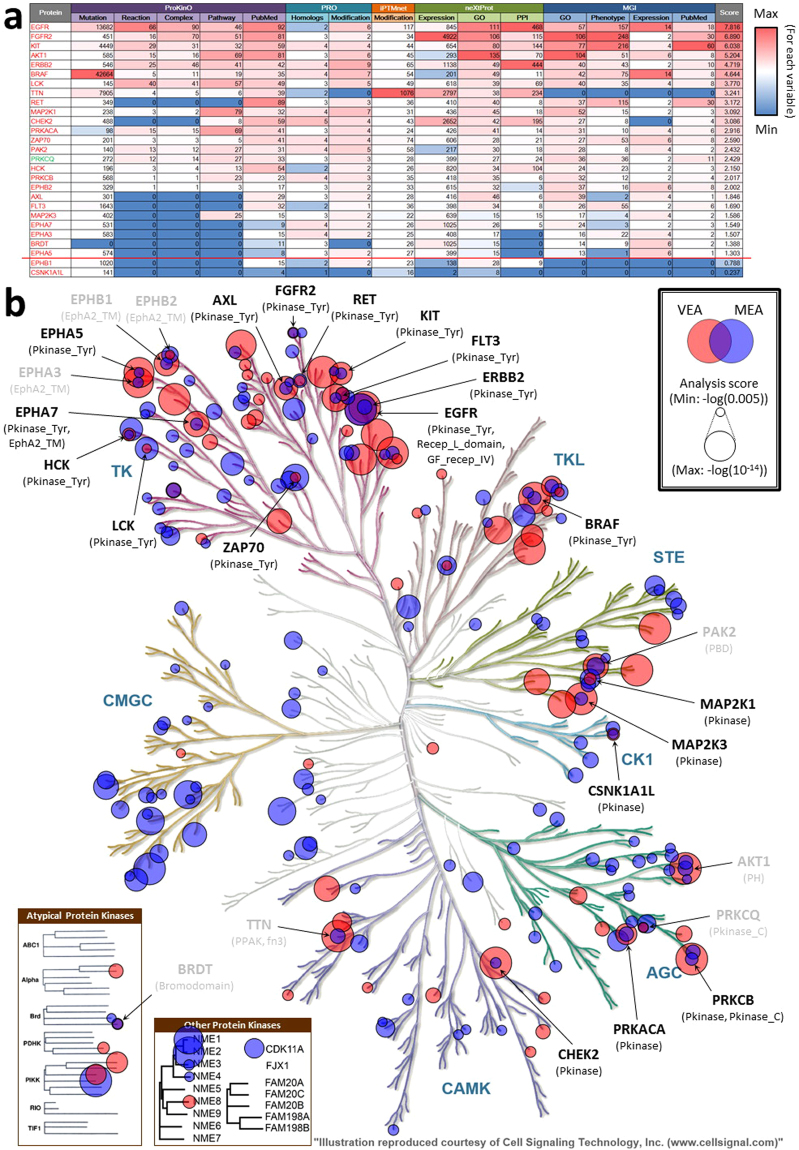
Annotation scores of protein kinases with enriched domains and visualization of the enriched domains in the human kinome tree. (**a**) Data volume, variety, and annotation score *ω* for each protein kinase with enriched VEA and MEA. The name of the protein kinase is shown in the first column in red if it is better-studied or green if under-studied; gradient colour (red for the maximum and blue for the minimum in the whole human kinome) indicates the data volume for each variable (column 2–16) and *ω* (last column). A threshold of high *ω* ( >1.00) is shown by a red line. (**b**) Protein kinases with functional domains with adjusted p-values in VEA or MEA less than 0.005 are represented by red and blue nodes, respectively; node size represents the score in VEA or MEA. Protein names are labelled only if any of their functional domains shows significant adjusted p-values in both VEA and MEA; black labels represent proteins with enriched protein kinase domains (either Pkinase or Pkinase_Tyr), while gray labels show proteins that only have other enriched domains; enriched domains are shown in parentheses. The human kinome tree was generated using KinMap^43^. Illustration is reproduced courtesy of Cell Signaling Technology, Inc. (http://www.cellsignal.comwww.cellsignal.com).

Most of the protein kinases with enriched domains (24/27) are better-studied and well-annotated (BS/High; Fig. 3a). However, the set includes one US/High protein kinase, PKCθ, which we analyse in detail below, and two BS/Low protein kinases, EPHB1 and CSNK1A1L. Given its association with cancer and the indication that there is a gap between available data and curated knowledge, further curation of EPHB1 and CSNK1A1L may be especially valuable.

### Classification of PTM and variant enriched kinases and domains

We mapped the 27 protein kinases with enriched domains onto the human kinome tree (Fig. 3b). The majority (12/19) of the enriched protein kinase domains fall within the tyrosine kinase group. Enriched regulatory domains are also observed in tyrosine kinases (e.g., growth factor receptor domain IV (GF_recep_IV) in EGFR). Tyrosine kinases can be classified as receptor tyrosine kinases (RTKs) or cytoplasmic tyrosine kinases (CTKs) depending on whether they contain membrane-spanning domains. Among the enriched domains in the tyrosine kinase group, 12 are found in 9 distinct RTKs, while none are from CTKs (Supplementary Data S1). The reason for this may be that many of the well-characterized RTK signalling circuits involve autophosphorylation of the RTK as opposed to trans-phosphorylation of a distinct substrate^17^; thus, loss of PTM sites in RTKs is more likely to disrupt signalling. There are several members of the STE group enriched in disease variants, but only three members, MAP2K1, MAP2K3, and PAK2, have functional domains enriched in PTMs as well. Conversely, the CMGC group has several members with domains enriched in PTMs, but not in cancer variants.

**Figure 4:**
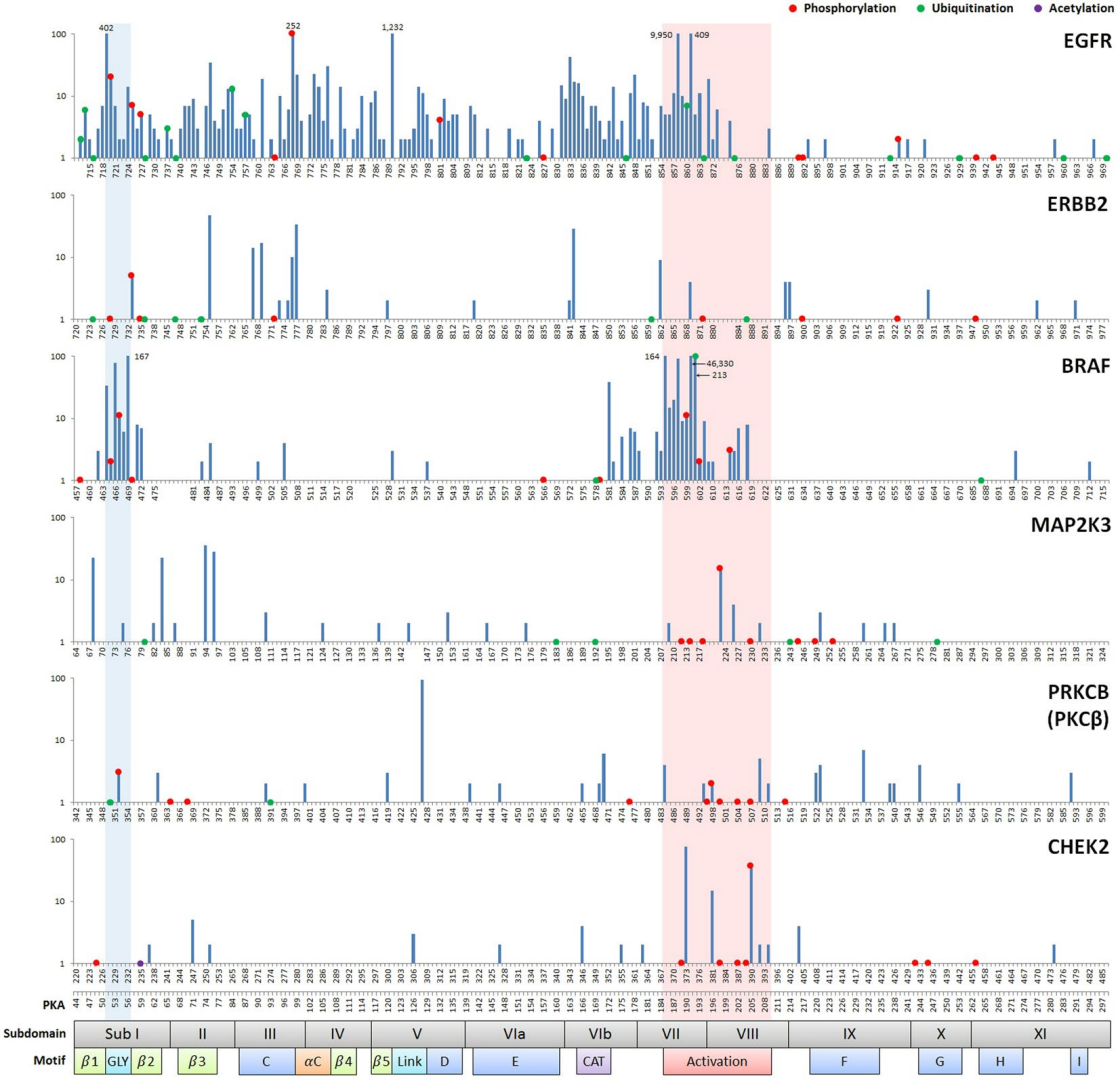
Variants and PTMs in the six most-enriched protein kinase domains. X-axis: sequence position and corresponding PKA position/subdomain/motif; y-axis: mutation frequency (logarithmic scale) across all cancer samples in COSMIC (exact frequency is labelled if it is greater than 100); red dot: phosphorylation; green dot: ubiquitination; purple dot: acetylation; dot on top of the bar: mutation-PTM overlapping site; Sub I~XI: subdomain I to XI; *β*1~5: beta 1 to beta 5 strands; C~I: C-helix to I-helix; GLY: glycine-rich loop; *α*C: alphaC-beta4 loop; Link: linker; CAT: catalytic loop; Activation: activation loop.

The mutation frequencies (across all the cancer samples in COSMIC) of all positions within the six most-enriched protein kinase domains are shown in Fig. 4. The glycine-rich loop (Fig. 4, blue highlighting) and the activation loop (Fig. 4, red highlighting) are hotspots for residues that are mutated and/or post-translationally modified. There are mutation-PTM overlapping sites within the glycine-rich loop of EGFR, BRAF, and PKCβ (phosphorylation sites S720, S465/S467, and S352, respectively) and within the activation loops of EGFR, BRAF, MAP2K3, PKCβ, and CHEK2 (ubiquitination sites K860/K875 of EGFR and K601 of BRAF; phosphorylation sites T599/S602/S602 of BRAF, T222/Y230 of MAP2K3, T498 of PKCβ, and Y390 of CHEK2). Because the glycine-rich loop is important for ATP binding and catalytic activity and the activation loop is important for kinase regulation, mutations in these PTM positions are expected to have a functional impact.

Hanahan and Weinberg have defined ten hallmarks, or critical biological capabilities, of tumour cells^18^. Using information from the COSMIC Cancer Gene Census, which has associated cancer-driver proteins with the hallmarks they affect, we investigated the hallmark profile of protein kinases. (Supplementary Fig. S2 and Supplementary Data S1). There are 36 protein kinases in the Cancer Gene Census; seven of them (EGFR, ERBB2, BRAF, FLT3, PRKACA, LCK, and FGFR2) had enriched PTM/mutation domains in our study. Of the 36 protein kinases in the census, 75% are associated with promoting proliferative signalling and 69% are associated with escaping programmed cell death. Notably, all seven enriched protein kinases have these hallmark properties. These two hallmarks, along with the ability to evade growth inhibitory signals comprise the survival hallmark network (defined by Wang *et al*.^19^) and underlie the fundamental ability of a cancer cell to over-proliferate and form a tumour.

### Case studies: variants, PTMs, and cancer

The protein kinase domains and mutation-PTM overlapping sites identified in the enrichment analysis are candidate regions where aberrant PTMs could contribute to cancer pathogenesis. To gain insight into mechanisms that could mediate the connection between PTM and cancer variants in these regions, we performed SPARQL federated queries of protein resources to obtain rich information at the protein, domain, variant, and PTM levels (Supplementary Fig. S1b). We then performed two case studies in which we selected enriched domains and sites and analysed the detailed query results with the goal of explaining how PTM disruption could result in tumorigenesis. We also performed cell-based studies to test the functional impact of mutational alterations of PTM sites in the selected protein kinases. Results of the federated queries that are relevant to the case studies are summarized in Table 2.

**Table 2:** Integrative annotation for case studies.

Gene	Position	Annotation	Variable	Value	Service
EGFR		Gene level	Cellular Component	Endosome membrane	neXtProt/MGI
				Plasma membrane	neXtProt/MGI
				basolateral plasma membrane	neXtProt/MGI
			Reaction	EGFR dimerization	ProKinO
			Complex	EGF:EGFR dimer [plasma membrane]	ProKinO
				L1-EGFR trans-heterodimer	PRO
			Pathway	Constitutive PI3K/AKT Signaling in Cancer	ProKinO
			Molecular Function	protein localization to nucleus	PRO
				Protein binding	neXtProt/MGI
				Protein phosphatase binding	neXtProt/MGI
			Biological Process	Positive regulation of cell proliferation	neXtProt/MGI
				epidermal growth factor receptor signaling pathway	neXtProt/MGI
			Phenotype	dilated respiratory conducting tubes	MGI
				respiratory distress	MGI
				abnormal lung interstitium morphology	MGI
				abnormal lung development	MGI
	S768	Residue level(General)	PKA Position	98	ProKinO
			Motif	N-lobe	ProKinO
				C_helix	ProKinO
				subdomainIII	ProKinO
		Residue level(Mutation)	Mutation Count	252	ProKinO
			Mutant Type (MT)	I	ProKinO/neXtProt
				G	ProKinO/neXtProt
			SNP	(MT = ’I’) rs397517108	neXtProt
				(MT = ’I’) rs121913465	neXtProt
				(MT = ’G’) rs756614898	neXtProt
			Mutation Description	(MT = ’I’) higher levels of basal autophosphorylation	neXtProt
		Residue level(PTM)	Proteoform	PR:000049851; S768-phosphorylated form; inhibitory effect on EGFR kinase activity	PRO
			Modification	Phosphorylation	PRO/iPTMnet
			Enzyme	CAMK2A	iPTMnet
			Equivalent Mouse Site	Q01279 S770: no evidence for modification	iPTMnet
PRKCB(PKCβ)		Gene level	Cellular Component	Cytoplasm	neXtProt/MGI
				Plasma membrane	neXtProt/MGI
			Reaction	PRKCB binds diacylglycerol and phosphatidylserine	ProKinO
			Complex	Activated PKC beta [plasma membrane]	ProKinO
			Pathway	Glioma	neXtProt
				Pathways in cancer	neXtProt
				VEGFR2 mediated cell proliferation	neXtProt
			Biological Process	Positive regulation of B cell receptor signaling pathway	neXtProt/MGI
				Positive regulation of I-kappaB kinase/NF-kappaB signaling	neXtProt/MGI
				Positive regulation of NF-kappaB transcription factor activity	neXtProt/MGI
				Positive regulation of vascular endothelial growth factor receptor signaling pathway	neXtProt/MGI
				Positive regulation of angiogenesis	neXtProt/MGI
	S661 (S660 in PKCβII)	Residue level(General)	Functional Domain	AGC-kinase C-terminal	neXtProt
		Residue level(Mutation)	Mutation Count	6	ProKinO
			Mutant Type (MT)	F	ProKinO
				C	ProKinO/neXtProt
		Residue level(PTM)	Proteoform	PR:000049877; S654 & S660- phosphorylated isoform-betaII; mouse	PRO
				PR:000049878; S660 & S664- phosphorylated isoform-betaII	PRO
				PR:000049879; S660 & S673- phosphorylated isoform-betaII; mouse	PRO
			Modification	Phosphorylation	PRO/iPTMnet
			Enzyme	PRKCB	iPTMnet
			Equivalent Mouse Site	P68404 Prkcb S660; phosphorylated	PRO/iPTMnet
	T642 & S661(T641 & S660 in PKCβII)	Residue level(PTM)	Proteoform	PR:000049855; T500, T641, S660-phosphorylated PRKCB-2; increased protein phosphorylation; increased localization to cytoplasm	PRO
			Modification	Phosphorylation	PRO
PRKCQ(PKCθ)		Gene level	Cellular Component	Cytoplasm	MGI
				Plasma membrane	MGI
			Reaction	Autophosphorylation of PKC-theta	ProKinO
			Complex	Active PKC theta bound to DAG [plasma membrane]	ProKinO
			Biological Process	Negative regulation of T cell apoptotic process	neXtProt/MGI
				Positive regulation of T cell activation	neXtProt/MGI
				Positive regulation of T cell proliferation	neXtProt/MGI
				Positive regulation of telomerase activity	neXtProt
				Positive regulation of telomere maintenance via telomerase	neXtProt
	S695	Residue level(General)	Functional Domain	AGC-kinase C-terminal	neXtProt
		Residue level(Mutation)	Mutation Count	1	ProKinO
			Mutant Type (MT)	F	ProKinO/neXtProt
		Residue level(PTM)	Proteoform	PR:000049857; S695-phosphorylated form; increased protein serine/threonine kinase activity	PRO
			Modification	Phosphorylation	PRO/iPTMnet
			Equivalent Mouse Site	Q02111 S695; phosphorylated	iPTMnet

### Case study 1: Inhibition of EGFR activity via phosphorylation of S768

We first focused on a mutation-PTM overlapping site, S768, in the tyrosine kinase domain of EGFR. We chose this example for several reasons: EGFR was the most enriched protein kinase in our analysis and exemplifies the tyrosine kinase domain, the most enriched domain in our dataset. EGFR also received the highest annotation score *ω* (7.81), and S768 was the most frequently mutated PTM site within the EGFR protein kinase domain (Fig. 4).

EGFR is involved in the epidermal growth factor signalling pathway and positively regulates cell proliferation in both human and mouse (Table 2, neXtProt/MGI). It is also involved in constitutive PI3K/AKT signalling in cancer (Table 2, ProKinO). Analysis of copy number variation data from COSMIC (Supplementary Methods) reveals that EGFR copy number gains significantly outnumber copy number losses in several tumour tissues (central nervous system, ovary, and pancreas; Supplementary Fig, S3 and Supplementary Data S1), consistent with an oncogenic, growth-promoting role for EGFR in cancer. There are 252 cancer-associated S768 mutations at EGFR (Table 2, ProKinO), and two SNPs that result in the substitution of isoleucine for serine at position 768 (rs397517108 and rs121913465, Table 2, neXtProt) are considered pathogenic or likely pathogenic in lung cancer, according to ClinVar^20^.

S768 is a phosphosite, and the S768-phosphorylated form of EGFR has reduced kinase activity (PR:000049851; Table 2, PRO). Mutations at this site change the serine to isoleucine or glycine, both of which would disrupt phosphorylation (Table 2, ProKinO/neXtProt), and the S768I mutation is associated with higher levels of basal autophosphorylation (Table 2, neXtProt). Taken together, the PTM and variant annotation support the idea that S768 phosphorylation is inhibitory to EGFR kinase activity.

**Figure 5:**
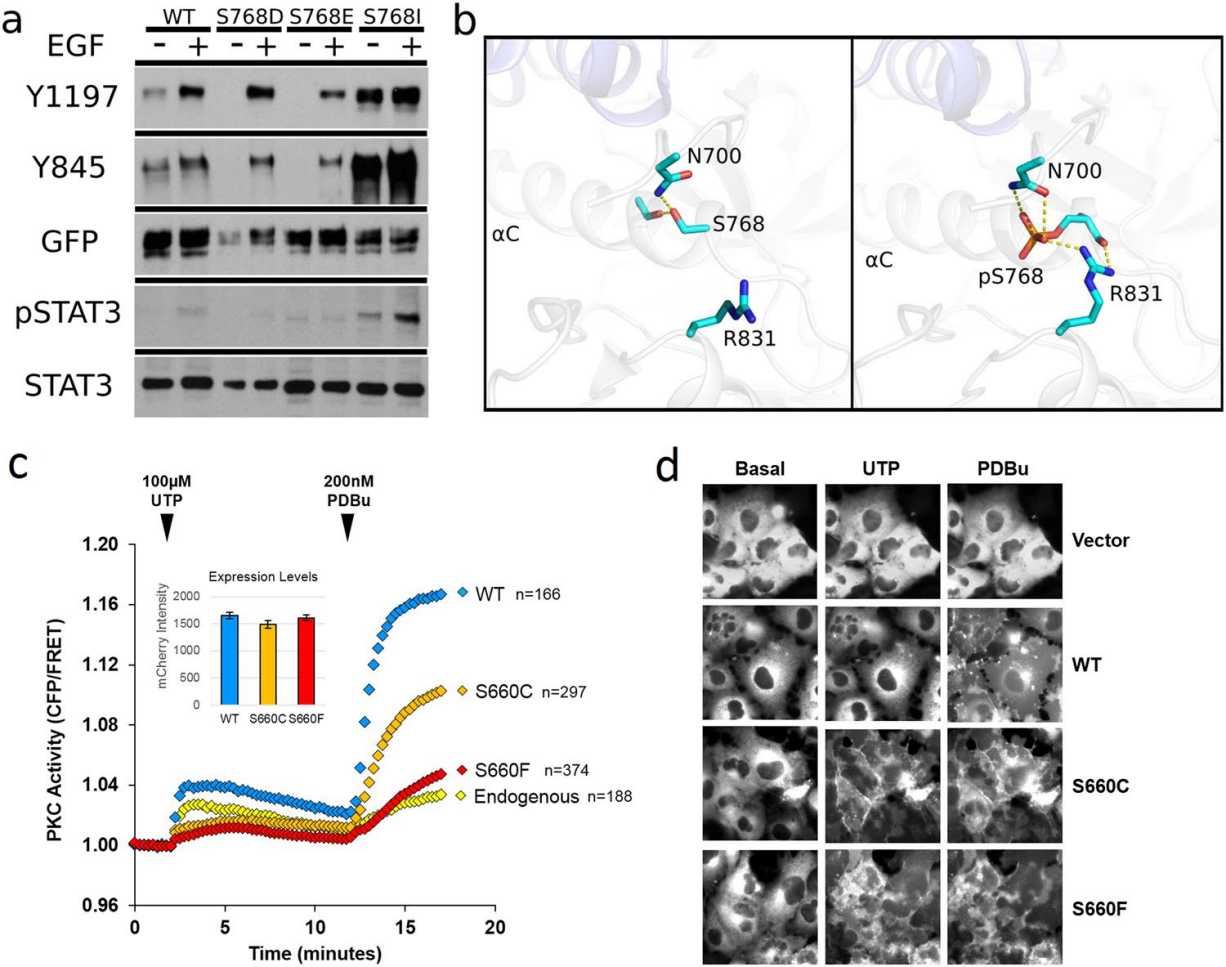
Biochemical impact of S768 phosphorylation in EGFR and S660 mutations in PKCβII. (**a**) Phospho-mimic mutations (S768D, S768E) and oncogenic mutation (S768I) alters the EGFR C-terminal tail (Y1197) and activation loop (Y845) phosphorylation. EGFR downstream signalling (STAT3) phosphorylation is also perturbed by the mutations. Each blot is obtained independently using designated antibody from the same cell lysis samples. An exposure time of 5 min is used for all blots. (**b**) Structural models comparing unphosphorylated/phosphorylated (S768) EGFR dimer. The 3D structures are represented by PyMOL^44^ (**c**) Normalized FRET-ratio changes (mean ± SEM) showing an agonist-induced activity of mCherry-tagged PKCβII wild-type (WT) or PKCβII hydrophobic (HF) motif mutants S660C and S660F in COS-7 cells co-expressing CKAR and treated with UTP (100 *μ*M), followed by PDBu (200 nM). mCherry intensity (inset) reflects expression levels of overexpressed PKC protein. Data are from three independent experiments. (**d**) Representative mCherry images of PKC translocation in COS-7 cells before agonist addition (Basal, t = 0 min), after UTP addition (UTP, t = 12 min), and after PDBu addition (PDBu, t = 17 min). mCherry vector was used as a negative control.

To test this hypothesis, we experimentally replaced the serine at position 768 with negatively charged residues (S768D and S768E), mimicking the constitutively phosphorylated state of EGFR. Consistent with our hypothesis, cell-based assays show that phosphomimetic mutations S768D and S768E decrease ligand-independent phosphorylation of both the activation loop (Y845) and the C-terminal tail (Y1197) compared to wild-type and oncogenic (S768I) EGFR (Fig. 5a). Downstream signalling (STAT3 activation) was also negatively affected by the phosphomimetic mutations (Fig. 5a), suggesting that phosphorylation of S768 inhibits EGFR activity.

S768 is located in the C-helix of subdomain III in the N-lobe of protein kinase domain (Table 2, ProKinO). Previous studies on EGFR have demonstrated that this region is part of an asymmetric dimer interface critical for kinase activation^21,22^. S768 positions the critical juxtamembrane (JM) segment of EGFR through a hydrogen bonding interaction with N700. Structural modelling suggests that S768 phosphorylation could impair kinase activity by altering the interactions in the receiver kinase interface and presumably hindering homodimerization (Fig. 5b). In contrast, the S768I variant cannot be phosphorylated and the hydrophobic nature of isoleucine may increase dimer stability, leading to the observed constitutive EGFR activity.

The aggregated annotation and our experimental data point to a model where the S768I mutation drives tumourigenesis by disrupting EGFR autophosphorylation and downstream signalling.

In mouse, Egfr mutations are associated with many respiratory phenotypes (Table 2, MGI), suggesting that mouse models of Egfr mutations may be relevant to lung diseases. Although the iPTMnet multiple sequence alignment shows that the position equivalent to S768 in human EGFR is conserved in mouse EGFR (human EGFR S768 aligns with mouse Egfr S770), there is no experimental phosphorylation information for this mouse site. It would be interesting to test whether this phosphorylation event and its consequences for EGFR activity are conserved in mouse.

### Case study 2: Role of protein kinase C terminal domain phosphorylation in the regulation of PKC family kinases

Our second study focused on the protein kinase C (PKC) terminal domain, also referred to as the C-terminal tail or AGC tail^23^ (Pkinase_C, Pfam ID: PF00433), which was PTM/mutation enriched in two members of the PKC family, PRKCB (PKCβ) and PRKCQ (PKCθ). We were interested in this region/domain because these protein kinases both contain a structurally conserved mutation-PTM overlapping site. Moreover, PKCθ is the only protein kinase in our enriched dataset that is considered under-studied by the NIH metric (Jensen PubMed score: 9.4, R01 count: 0, and PubTator score: 96.0). Interestingly, PKCθ was in the top 25% of protein kinases according to our annotation score *ω* (score of 2.4), suggesting that we could use the aggregated annotation results to gain insight into this protein kinase, despite its status as under-studied. PKCβ had a similar annotation score (2.0) but is considered better-studied by the NIH metric (Jensen PubMed score: 83.5, R01 count: 126, and PubTator score: 383.8).

**Figure 6:**
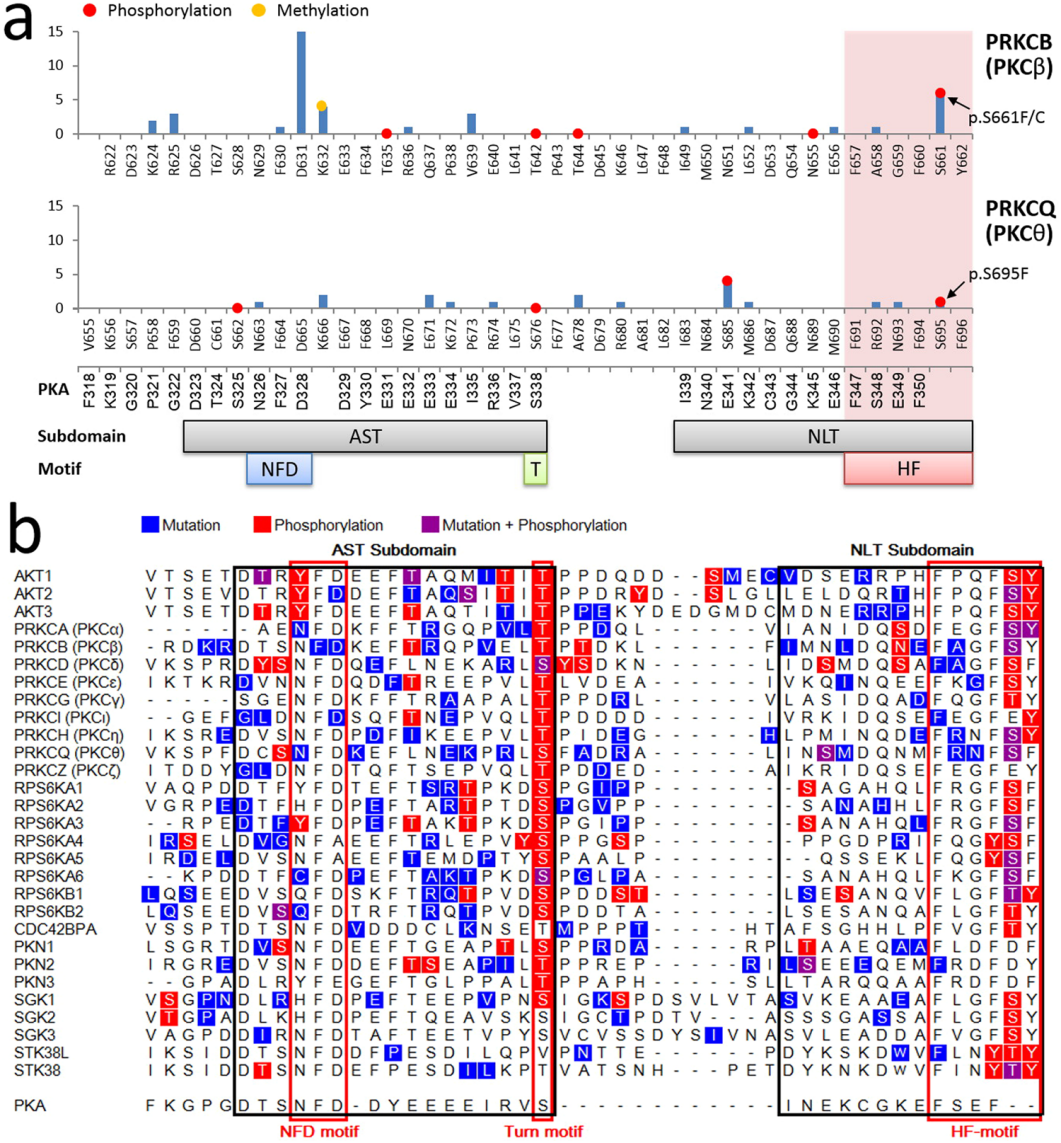
Mutation and PTM sites in the C terminal tail domain of AGC kinases. (**a**) Mutation and PTM sites in enriched protein kinase C terminal tail domains. X-axis: sequence position and corresponding PKA position/subdomain/motif; y-axis: mutation frequency across all cancer samples in COSMIC; red dot: phosphorylation; yellow dot: methylation; dot on top of the bar: mutation-PTM overlapping site; AST: active-site tether subdomain; NLT: N-lobe tether subdomain; NFD: NFD motif; T: turn motif; HF: hydrophobic motif. (**b**) Mutation and phosphorylation sites in all protein kinase C terminal domains. Sequences of all protein kinase C terminal tail domains defined by Pfam are aligned to PKA sequence by MAFFT alignment; black rectangles: subdomains; red rectangles: motifs; residues marked in blue: mutation sites (frequency is ignored); residues marked in red: phosphorylation sites; residues marked in purple: mutation-phosphorylation overlapping sites.

The mutation-PTM overlapping site with the greatest number of cancer-associated mutations in the Pkinase_C domain of PKCβ is S661, which lies in the HF-motif (Fig. 6a). This site corresponds to S660 in PKCβ isoform 2 (PKCβII; PR:000049855; Table 2, PRO), and S695 in PKCθ (PR:000049857; Table 2, PRO), which is also a mutation-PTM overlapping site. Moreover, this site is a hotspot of mutation-phosphorylation overlapping sites across most AGC kinases (Fig. 6b). All the canonical phosphorylatable residues (serine, threonine, and tyrosine) at this site are phosphorylation targets, and 10 out of these 24 residues (42%) are also mutation sites. Thus, we performed low-level federated queries for PKCβ and PKCθ to provide functional context for cancer mutations that alter C-terminal tail phosphorylation.

The HF-motif of PKC isozymes is autophosphorylated shortly after biosynthesis to yield a stable, autoinhibited species that is degradation- and phosphatase-resistant species. When phosphorylated at this key processing site, both protein kinases are primarily cytosolic and translocate to the membrane following agonist-dependent generation of diacylglycerol (Table 2, neXtProt/MGI). Kinase-inactive enzymes do not incorporate phosphate at this site and are shunted for degradation. Thus, mutations that disrupt phosphorylation would be predicted to be loss-of-function and support a tumour suppressive role of PKC.

Functional analysis of approximately 50 cancer-associated mutations in eight different PKC family members, including PKCβ, indicated that most of the mutations are loss-of-function, reducing or eliminating kinase activity^24^; evolutionary analysis performed by KinView^25^, a sequence visualization tool embedded in ProKinO, predicted and experimentally validated a PKCβ loss-of-function mutation D523N in the F-helix, although it is not a mutation-PTM overlapping site (Fig. 4). Correction of a cancer-associated inactivating mutation in PKCβ in a colon cancer cell line suppressed tumour formation in a xenograft model, supporting a tumour suppressor role for this isozyme. Consistent with this, higher levels of PKCβII protein in the normal distal epithelia of patients with colon cancer is associated with better prognosis^26^. Immunohistological studies annotated in the human cancer transcriptome^27^ (www.proteinatlas.org/pathology) reveal that high PKCβII protein expression confers an increased survival outcome in colon, breast, prostate, lung, and liver cancer, consistent with a tumour suppressor function. Copy number variation analysis indicates that the copy number for PKCβ and PKCθ varies depending on cancer type (Supplementary Fig. S3). In breast and ovarian cancers, PKCβ copy number amplifications exceed copy number losses, however, it is unclear how this relates to protein expression. In contrast, in pancreatic tumours of the carcinoid-endocrine subtype, copy number losses are higher than copy number amplifications. Similarly, in skin tumours, copy number data suggests PKCθ is a tumour suppressor. The PKCβII S660 mutations in our dataset were all detected in skin cancers, mainly melanomas. Unfortunately, there are too few PKCβ copy number variants in melanomas to determine whether there is a significant difference in copy number gains vs. losses in this cancer subtype.

To assess the effects of the cancer-associated PKC HF-motif mutants on PKC activity, we used the C-Kinase Activity Reporter^28^ to measure agonist-evoked PKC kinase activity in live cells (Fig. 5c). mCherry-tagged hPKCβII wild-type or mutant constructs were overexpressed in COS-7 cells co-expressing CKAR and stimulated with uridine 5’ triphosphate (UTP), a purinergic agonist that elevates the second messengers Ca^2+^ and diacylglycerol to reversibly activate PKC; they were also treated with PDBu to maximally activate the PKC^28^. Compared to wild-type PKCβII, PKCβII S660C and S660F mutants displayed impaired response to both UTP and PDBu (Fig. 5c). Additionally, the mutants had enhanced plasma membrane translocation indicative of an inability to autoinhibit and mask the membrane-targeting domains^29^: whereas membrane translocation of wild-type PKCβII was observed only following PDBu stimulation, the weaker stimulation by UTP resulted in readily observable translocation of the mutants (Fig. 5d).

The reduction of activity observed for the HF-motif mutants indicates that they are loss-of-function. Furthermore, agonist-evoked activity in cells expressing the HF-motif mutants was reduced below endogenous levels, suggesting that these enzymes possess a dominant-negative function and are suppressing the activity of endogenous PKC. Additionally, the HF-motif mutants exhibited enhanced membrane translocation upon stimulation with physiological agonist, indicating that they adopt an unprimed, open conformation with exposed membrane targeting domains^29^. Thus, the HF-motif is required for the conformational rearrangement that masks the membrane targeting domains and allows PKC to adopt the catalytically competent, but autoinhibited conformation.

## Discussion

Many biological studies, including proteome-wide analyses and detailed experiments on individual proteins, benefit from drawing information from multiple resources. Therefore, it is critical to develop methods to facilitate data integration. In this study, we have taken advantage of an RDF representation of information that can be queried using SPARQL. Specifically, we integrated information of various levels of granularity, including protein/gene level information from neXtProt and MGI, protein kinase family information from ProKinO, and PTM and variant-site level information from ProKinO, PRO, and iPTMnet, to explore protein kinase PTMs in the context of cancer. This approach allowed us to take advantage of the expertise invested in each resource and to conduct a more detailed analysis than would be possible with any single resource.

Using the aggregated data, we performed two kinome-wide studies: we developed a metric to assess the quantity of annotation available about each protein kinase in curated resources and we identified functional domains in protein kinases that are statistically enriched in cancer-associated mutations and PTMs. Based on the outcome of these analyses, we selected two cancer-associated PTMs for in-depth study: S768, located in the protein kinase domain of EGFR, and S661/S695 located in the PKC terminal domains of PKCβ and PKCθ, respectively. Our annotation score *ω* revealed 108 protein kinases that are relatively well-studied according to their profile in the literature but are sparsely annotated, suggesting that it would be valuable to prioritize curation efforts for these protein kinases. We also found 34 protein kinases that are considered under-studied in the scientific literature but are in fact quite richly annotated in bioinformatics resources. One of the protein kinases chosen for in-depth study, PKCθ, was in this category, and the available annotation was sufficient to propose mechanisms for the oncogenic effect of PTM site mutations in this protein. Our analysis identified 32 domains in 27 protein kinases that are statistically enriched in cancer-associated mutations and PTMs. The dual enrichment suggests that disruption in PTM in these domains may contribute to oncogenesis. This is supported by our in-depth study of EGFR S768. Review of the integrated data for this site suggested that mutations that lead to loss of phosphorylation at this site would result in an abnormally active, potentially oncogenic kinase. We performed experiments using phosphomimetic mutations at this position as well as structural modelling to confirm that phosphorylation is likely to reduce kinase activity. In our analysis of the Pkinase_C domains of PKCβ and PKCθ, we found that loss of HF-motif phosphorylation not only inactivates the kinase but results in a dominant-negative effect with respect to global PKC signalling in the cell. Our experimental studies indicate that PKCβII HF-motif phosphorylation site mutants are loss-of-function, supporting clinical studies and cellular studies indicating PKCβ is a tumour suppressor; thus, failure to phosphorylate the HF-motif may contribute to disease progression by impairing PKC activity.

Hundreds of RDF data resources, including nearly 100 in the life sciences are available within the Linked Open Data^30^ (LOD) initiative. Recently, a federated query utility has been added to SPARQL, enabling aggregate queries from distributed RDF resources that, otherwise, would be very difficult to obtain. In practice, however, formulating such integrative SPARQL federated queries is not an easy task. To write such queries, a researcher must (1) fully understand the organization (schema) and the contents of all of the involved RDF datasets, (2) be familiar with the precise syntax and semantics of SPARQL and federated queries, and (3) be able to perform any additional data analytics tasks on the retrieved results. Recently, the first problem has been partially addressed by a system called BioFed^31^, which helps in selecting the data sources and automatically re-writing the query as a federated query. However, the users must still formulate the initial SPARQL query, and many biologists are not familiar with the SPARQL syntax and semantics. To address the second problem, we have created SPARQLing^32^, a graphical SPARQL query formulation system designed for easy query formulation by people not familiar with SPARQL. We are planning to extend SPARQLing to access remote, distributed data sources. Furthermore, we are planning to integrate SPARQLing with statistical analysis software such as R^33^ for performing post-query analysis on the returned results. Another data integration challenge arises when studying individual amino acid positions within a protein. It is often difficult to identify the equivalent site across isoforms and orthologs because of differences in position numbering. ProKinO has addressed this issue for protein kinases by developing KinView, a tool that, among other functions, maps protein kinase sequences to a reference protein kinase A (PKA) sequence^25^. Likewise, to facilitate conservation studies of PTM and variant sites, PRO is currently developing a sub-ontology for sites. This ontology will define classes of positions within proteins; equivalent positions in isoforms or orthologs can then be related through belonging to the same position class.

In conclusion, by integrating protein-related data from multiple sources, we explored current knowledge gaps in the functional coverage of the human kinome, and the relationships connecting kinome cancer variants and PTMs. We identified protein kinases in need of further curation as well as kinase functional domains where aberrant PTM may contribute to disease; furthermore, we proposed testable hypotheses about cancer variants altering key PTM sites and experimentally tested the hypotheses in EGFR and PKCβ. Continued development to improve automated information retrieval across resources is necessary to enable this approach to be used on a larger scale, and particularly to allow biologists lacking an informatics background to fully make use of these invaluable resources.

## Methods

### Variant enrichment analysis

Domains enriched in disease variants were identified using a statistical test: Variant Enrichment Analysis (VEA), which examines the frequency of mutations observed in functional domain *d* across different cancer samples. In VEA, we refer to a recent study^34^ and assume that mutation distribution in functional domains follows a Poisson distribution. Given this assumption, the expected number of mutations for each domain *d* is defined as:

1 $${E}_{d}=\sum _{a}{E}_{a}{N}_{ad,}$$

where *N*_*ad*_ is the number of amino acid *a* present in domain *d*, and the average number of mutations per amino acid *a*, *E*_*a*_, is given by:

2 $${E}_{a}=\frac{{O}_{a}}{{N}_{a}},$$

where *O*_*a*_ is the number of mutations of amino acid *a* present in the whole human kinome across different samples, and *N*_*a*_ is the total number of amino acid *a* present in the whole human kinome. For each domain *d*, the parameter *λ* of the Poisson distribution was estimated by *E*_*d*_. The significance (an upper tail p-value) of this enrichment analysis for each domain *d* was generated by comparing the exact number of mutations observed in domain *d* with the null distribution. Adjusted p-values of all functional domains in VEA were generated under false discovery rate (FDR) control by Benjamini-Hochberg procedure^35^ (significant level: 0.005). The score of a domain in VEA is defined as the negative log-transformation of the adjusted p-value (if the adjusted p-value is less than 10^−14^, it is replaced by 10^−14^).

Cancer variants and protein functional domains were obtained from COSMIC (v81) and Pfam (release 30.0), respectively. In total, there are 217,925 non-redundant protein kinase missense mutations across all cancer samples. To avoid potential bias in enrichment analyses we considered variants only from genome-wide screens (92,222 missense mutations remained). Protein kinase mutations in COSMIC were mapped to specific positions in the canonical UniProtKB sequence using the MAFFT alignment program^36^. This mapping resulted in 66,955 non-redundant missense substitutions located in 1,633 distinct functional domains in the human kinome and the rest 25,267 mutations located in low-complexity or disorder regions.

### Modification enrichment analysis

Domains enriched in post-transcriptional modifications (PTMs) were identified using the Modification Enrichment Analysis (MEA), which examines the number of residues modified within functional domain *d*. We assume the occurrence of a PTM event in each type of residue follows is an independent Bernoulli trial. Because the occurrence probabilities of all types of residue are non-identical, the number of residues modified within a specified protein sequence interval, a functional domain, follows Poisson’s binomial distribution, which can be approximated to Poisson distribution by Poisson approximation method^37,38^. The expected number of residues modified within functional domain *d* is defined as *E*_*d*_ by the same formulae (1) and (2) with a different definition in *O*_*a*_, which is defined as the number of modified residues of amino acid *a* present in the whole human kinome in MEA. The probability *p* of the Bernoulli trial for amino acid *a* is equal to *E*_*a*_, while the parameter *λ* of the approximated Poisson distribution for domain *d* is estimated by *E*_*d*_. For each domain *d*, we calculated the significance (an upper tail p-value) of this enrichment analysis by comparing the observed number of residues modified within domain *d* with the null distribution. Adjusted p-values of all functional domains in MEA were generated under FDR control by Benjamini-Hochberg procedure (significant level: 0.005). The score of a domain in MEA is also defined as the negative log-transformation of the adjusted p-value (if the adjusted p-value is less than 10^−14^, it is replaced by 10^−14^).

PTMs with their confidence scores were obtained from iPTMnet. Only manually curated PTMs with confidence scores greater than 1 were used in this study. This resulted in 16,837 PTMs mapping to 16,545 unique positions in the human kinome.

### SPARQL federated query

Federated query is a technique for executing a distributed SPARQL query against multiple data sets available from different SPARQL endpoints. In this study, SPARQL federated queries were used to analyse the human kinome at both high and low levels. The high-level federated queries probed the data volume and variety of the human kinome (Fig. 2d and Supplementary Data S1), while the low-level federated queries integrated a variety of annotations for the protein kinases, shown in the two case studies (Table 2).

The pseudocode of the high-level federated query is shown in Supplementary Fig. S1a (all executable queries are available at https://github.com/esbg/SPARQL). The query calculated the number of records (values) of different types of annotations (variables) from different resources (service providers) for each protein kinase. Fourteen variables were selected from four service providers: Mutation, Reaction, Complex, Pathway, and PubMed (human) were retrieved from ProKinO (v2.0); Homologs and Modifications were obtained from PRO (v52.0); Expression (human), GO (human Gene Ontology), and PPI (protein-protein interaction) data were retrieved from neXtProt (v2.9.0); GO (mouse Gene Ontology), Phenotype, Expression (mouse), and PubMed (mouse) were retrieved from MGI dataset via Bio2RDF^39^ (v3.0). In addition to the number of modifications obtained from PRO by a SPARQL query, Fig. 3a and Supplementary Data S1 also show the number of modifications extracted directly from iPTMnet’s flat files (v4.1). The annotation score of protein kinase *i*, *ω*_*i*_, is defined as the sum of the normalized values of all variables *j*:

3 $${\omega }_{i}=\sum _{j}\frac{{\nu }_{ij}}{max({\nu }_{j})},$$

where *v*_*ij*_ is the value of variable *j* for protein kinase *i*, and *v*_*j*_ represents all the values of variable *j* for all protein kinases. A threshold for annotation score, 1.00, is to delineate high vs. low annotation scores.

The pseudocode of the low-level federated query is shown in Supplementary Fig. S1b. It collected the values of selected variables from the four service providers. Table 2 only show the variables described in the two case studies. For variables with multiple values, only the values with the highest number of annotations or evidence are shown. iPTMnet does not provide a SPARQL endpoint, so to preserve analysis integrity, annotations from iPTMnet were manually collected and shown in Table 2.

### EGFR S768 mutation analysis

#### Mutagenesis

The procedure for EGFR mutational analysis is described in our previous studies^40,41^. Briefly, desired point mutations (S768D, S768E, S768I) were introduced into a pEGFP-N1-EGFR plasmid using QuikChange Site-Directed Mutagenesis Kits (Agilent Genomics, CA, USA). Mutant plasmids were purified and confirmed via DNA sequencing.

#### Cell transfection and western blot analysis

Chinese Hamster Ovary (CHO) cells grown in high-glucose Dulbecco’s modified Eagle medium (DMEM) (Cellgro, Manassas, VA, USA) with 10% fetal bovine serum (FBS) (Bioexpress, UT, USA) to approximately 30% confluency. Lipofectamine-2000 (Thermo Fisher Scientific, Waltham, MA, USA) was used to transfect wild-type/mutant plasmids (5 *μ*g DNA per 30 mm plate) into CHO cells by following the manufacturer’s protocol. After 24 hours, GFP signals of the cells were inspected under a microscope for the transfection efficacy. CHO cells were then washed with phosphate buffered saline (PBS) (Cellgro, Manassas, VA, USA) and serum-starved in Ham’s F-12 media with L-glutamine (Cellgro, Manassas, VA, USA) for 18 h followed by EGF stimulation (100 ng/mL) (Sigma, St. Louis, MO, USA) for 5 min. CHO cells population was pooled in lysis buffer (50 mM Tris HCl pH 7.4, 1 mM EDTA, 150 mM NaCl, 1% Triton X-100, and 10% glycerol) at 4 °C. 150 *μ*l of total cell lyse were mixed with 50 *μ*l 4X SDS PAGE sample buffer (40% glycerol, 8% SDS, 200 mM Tris-HCl PH 6.8, 0.4% bromophenol blue, and 10% 2-mercaptoethanol) and heated at 95 °C for 7 minutes. The protein sample was resolved using 8% polyacrylamide gels and transferred to polyvinylidene fluoride (PVDF) membrane for western blot analysis. Membrane was blocked in 4% BSA-TBST buffer (4% bovine serum albumin, 5 mM Tris, 15 mM NaCl, 0.1% Tween 20, PH 8.0). The EGFR expression and autophosphorylation signal were probed using anti-GFP, anti-Y845-EGFR, anti-Y1197-EGFR antibodies (Cell Signaling, MA, USA). Downstream STAT3 signalling was probed using anti-Y705-STAT3, and anti-STAT3 antibodies (Cell Signaling, MA, USA). The membrane was visualized using Pierce ECL substrate (Thermo Scientific, MA, USA) for 5 minutes.

#### Equipment and settings

The digital images were scanned using EPSON perfection 4490 photo scanner at 300 dpi resolution. All resulting images were converted to black and white using Inkscape. The reported images are neither cropped nor enhanced by any means of post-processing.

### PKCβII S660 mutation analysis

#### Plasmid constructs and reagents

The C Kinase Activity Reporter (CKAR) was previously described^28^. mCherry-tagged constructs were cloned into pcDNA3 with mCherry at the N-terminus at the BamHI and XbaI sites. All mutants were generated by QuikChange II Site-Directed Mutagenesis (Agilent). Phorbol 12,13-dibutyrate (PDBu) and uridine-5’-triphosphate (UTP) were purchased from Sigma-Aldrich.

#### Cell culture and transfection

COS-7 cells were cultured in DMEM (Corning) containing 10% fetal bovine serum (Atlanta Biologicals) and 1% penicillin/streptomycin (GIBCO) at 37 °C in 5% CO_2_. Transient transfection was carried out using the Lipofectamine 3000 Transfection Reagent (Thermo Fisher Scientific).

#### FRET imaging and analysis

Cells were plated onto glass coverslips in 35 mm dishes, transfected with the indicated constructs, and imaged in Hanks’ balanced salt solution supplemented with 1 mM CaCl_2_. CFP, YFP, and FRET images were acquired with a 403 objective with a Zeiss Axiovert microscope (Carl Zeiss Microimaging) using an iXon Ultra EMCCD camera (Andor Technology) controlled by MetaFluor software version 7.10.1.161 (Universal Imaging) as described previously^42^. For activity experiments, COS-7 cells were co-transfected with the indicated mCherry-tagged PKC construct and CKAR. Baseline images were acquired every 15 s for 2 min before ligand addition. Förster resonance energy transfer (FRET) ratios represent CFP/FRET mean ± SEM from at least three independent experiments. All data were normalized to the baseline FRET ratio of each individual cell.

## Electronic supplementary material

Supplementary

Supplementary Data S1
