# Supplementary material for: Integrative annotation and knowledge discovery of kinase post-translational modifications and cancer-associated mutations through federated protein ontologies and resources
Source: Sci Rep. 2018 Apr 25;8:6518. doi: 10.1038/s41598-018-24457-1 (PMC5916945; doi:10.1038/s41598-018-24457-1)
Supplement: Supplementary file 1 — Supplementary [file 41598_2018_24457_MOESM1_ESM.pdf]

Integrative annotation and knowledge discovery of kinase post-translational modifications and cancer-associated mutations through federated protein ontologies and resources

Liang-Chin Huang[1,+], Karen E. Ross[2,+], Timothy R. Baffi[3], Harold Drabkin[4], Krzysztof J. Kochut[5], Zheng Ruan[1], Peter D'Eustachio[6], Daniel McSkimming[7], Cecilia Arighi[8], Chuming Chen[8], Darren A. Natale[2], Cynthia Smith[4], Pascale Gaudet[9], Alexandra C. Newton[3], Cathy Wu[2,8], and Natarajan Kannan[1,*]

[1]Institute of Bioinformatics, University of Georgia, Athens, GA, 30602, USA
[2]Protein Information Resource (PIR), Department of Biochemistry and Molecular & Cellular Biology, Georgetown University Medical Center, Washington, DC, 20007, USA
[3]Department of Pharmacology, University of California, San Diego, La Jolla, CA, 92093, USA
[4]The Jackson Laboratory, Bar Harbor, ME, 04609, USA
[5]Department of Computer Science, University of Georgia, Athens, GA, 30602, USA
[6]Department of Biochemistry & Molecular Pharmacology, NYU School of Medicine, New York, NY, 10016, USA
[7]Genome, Environment, and Microbiome (GEM) Center of Excellence, University at Buffalo, Buffalo, NY, 14203, USA
[8]Center for Bioinformatics and Computational Biology, University of Delaware, Newark, DE, 19711, USA
[9]SIB Swiss Institute of Bioinformatics, Lausanne, 1015, Switzerland
[*]nkannan@uga.edu
[+]these authors contributed equally to this work

Supplementary Methods

Copy number variation analysis

We used copy number variation to predict potential oncogenic or tumour suppressor roles for genes, as used in previous studies[1,2]. However, a gene does not always play the same role in different cancer types. For example, bilateral roles of a centromere protein in skin carcinogenesis are well documented[3]. Moreover, PKC isoforms can stimulate melanogenesis, suppress growth, or both (from different studies), in melanoma cells[4]. Genes' bilateral roles in different cancer types are identified and curated over time with several genes annotated as both oncogenes and TSGs in COSMIC Cancer Gene Census[5] v83 (October 2017). Therefore, the CNV analysis in this study is only to objectively identify significant amplification or deletion of protein kinases in different cancer subtypes without inferring its oncogenic or tumor suppressor role.

CNV data, including the data from International Cancer Genome Consortium (ICGC), The Cancer Genome Atlas (TCGA), and COSMIC Cell Lines Project, were obtained from COSMIC (v81). Whether a gene is amplified (Gain) or deleted (Loss) in a cancer sample is defined based on the original ICGC data, and the programs ASCAT 2.4[6] and PICNIC[7]. To avoid potential bias in CNV analysis we considered the variants only from genome-wide screens. Different transcript duplicates from the same gene in the same sample were removed. Inconsistent CNV types (Gain/Loss) of a gene in the same sample, due to inconsistencies in data from different sources, were also removed. The statistic we used for each gene i in cancer subtype j is shown below:

$$\delta_{ij} = \frac{G_{ij} - L_{ij}}{N_j} \tag{1}$$

, where δ_{ij} ranges from -1 to 1, G_{ij} is the number of cancer subtype j in which gene i is amplified (Gain), L_{ij} is the number of cancer subtype j in which gene i is deleted (Loss), and N_j is the sample size of cancer subtype j. In the CNV analysis,

cancer subtype j was determined by four descriptors given by COSMIC: primary site, site subtype 1, primary histology, and histology subtype 1. δ_{ij} of all the genes, except for protein kinases, in cancer subtype j with the same primary site were pooled to build null distributions. An upper tail p-value for determining the significance of amplification and a lower tail p-value for determining the significance of deletion for each protein kinase i in cancer subtype j were generated by comparing δ_{ij} with the null distribution of corresponding primary site. The following constraints were used to reduce the statistical error caused by sample bias. When building the null distributions, we required at least 50 samples for each cancer subtype ($N_j \geqslant 50$), and more than 500 samples for each primary site. To determine the significance of amplification/deletion for each protein kinase i in cancer subtype j, we again required 50 samples for the cancer type ($N_j \geqslant 50$), at least 15 of which must show copy number gain/loss ($G_{ij} \geqslant 15$ or $L_{ij} \geqslant 15$), and the absolute value of the difference ratio must differ from 0 by a minimal amount ($|\delta_{ij}| > 0.15$). P-values less than 0.05 were considered significant.

CNV analysis results along with null distribution (gray dots) and known oncogenes (red dots) and TSGs (green dots) of corresponding primary sites are plotted in Figure S3. Known oncogenes and TSGs in specific cancer types are defined by COSMIC Cancer Gene Census[5] only if their mutation type was “A” (amplification) for oncogenes or “D” (large deletion) for TSGs (Supplementary Data S1). Figure S3 shows that oncogenes and TSGs generally have positive and negative δ, respectively; exceptions may be from specific cancer subtype. For example, ERBB2 is known to be an oncogene and amplified in ovarian carcinomas[8,9], however, it is amplified (Gain) in 3 samples and deleted (Loss) in 111 samples out of the 115 ovarian carcinoma samples from Australian Ovarian Cancer Study (AOCS) (the red dot near the bottom (δ: -0.94) of the null distribution of ovarian cancer in Figure S3; primary site: ovary; site subtype 1: NS; primary histology: carcinoma; histology subtype 1: mixed adenosquamous carcinoma).

References

1. Zack, T. I. *et al.* Pan-cancer patterns of somatic copy number alteration. *Nat Genet.* **45**, 1134–40 (2013).
2. Wrzeszczynski, K. O. *et al.* Identification of tumor suppressors and oncogenes from genomic and epigenetic features in ovarian cancer. *PLoS One* **6**, e28503 (2011).
3. Okumura, K. *et al.* Cenp-r acts bilaterally as a tumor suppressor and as an oncogene in the two-stage skin carcinogenesis model. *Cancer Sci* **108**, 2142–2148 (2017).
4. Oka, M. & Kikkawa, U. Protein kinase c in melanoma. *Cancer Metastasis Rev* **24**, 287–300 (2005).
5. Futreal, P. A. *et al.* A census of human cancer genes. *Nat Rev Cancer* **4**, 177–83 (2004).
6. Van Loo, P. *et al.* Allele-specific copy number analysis of tumors. *Proc Natl Acad Sci U S A* **107**, 16910–5 (2010).
7. Greenman, C. D. *et al.* Picnic: an algorithm to predict absolute allelic copy number variation with microarray cancer data. *Biostat.* **11**, 164–75 (2010).
8. Tuefferd, M. *et al.* Her2 status in ovarian carcinomas: a multicenter gineco study of 320 patients. *PLoS One* **2**, e1138 (2007).
9. McAlpine, J. N. *et al.* Her2 overexpression and amplification is present in a subset of ovarian mucinous carcinomas and can be targeted with trastuzumab therapy. *BMC Cancer* **9**, 433 (2009).

Gene	Domain	Position	PKA	#Mutation	MutationAA	PTM
AKT1	PH	14		1	p.K14I/N	Acetylation
						Methylation
						Ubiquitination
		65		1	p.T65M	Phosphorylation
AXL	Pkinase_Tyr	724	214	3	p.R724C	Methylation
		726	216	1	p.Y726H	Phosphorylation
BRAF	Pkinase_Tyr	465	52	2	p.S465F	Phosphorylation
		467	54	11	p.S467L/F	Phosphorylation
		599	190	11	p.T599R/I/S	Phosphorylation
		601	192	213	p.K601E/Q/N/I/T/L/R	Ubiquitination
		602	193	2	p.S602Y/T	Phosphorylation
		605		10	p.S605R/G/F/N	Phosphorylation
		614	200	3	p.S614P	Phosphorylation
		671		2	p.R671Q	Methylation
BRDT	Bromodomain	331		1	p.Y331C	Phosphorylation
CHEK2	Pkinase	235	59	1	p.K235Q	Acetylation
		372	189	1	p.S372C	Phosphorylation
		390	205	37	p.Y390C	Phosphorylation
CSNK1A1L	Pkinase	146	177	2	p.T146N/A	Phosphorylation
		206	224	1	p.S206F	Phosphorylation
EGFR	Recep_L_domain	117		1	p.Y117C	Phosphorylation
	Pkinase_Tyr	713	45	2	p.K713F	Ubiquitination
		714	46	6	p.K714N/T	Ubiquitination
		716	48	1	p.K716R	Ubiquitination
		720	52	20	p.S720F/C/P/T	Phosphorylation
		725	57	7	p.T725M/C/A	Phosphorylation
		727	59	5	p.Y727C/H	Phosphorylation
		737	65	3	p.K737G/T/E	Ubiquitination
		752		7	p.S752F/P/Y	Phosphorylation
		754	84	13	p.K754E/Q/I/A/R	Ubiquitination
		757	87	5	p.K757R/N/M	Ubiquitination
		764	94	1	p.Y764S	Phosphorylation
		768	98	252	p.S768I/T/C/G/N/V	Phosphorylation
		801	132	4	p.Y801H/C	Phosphorylation
		846	176	1	p.K846R	Ubiquitination
		860	190	7	p.K860I/E	Ubiquitination
		869		1	p.Y869C	Phosphorylation
		875	201	1	p.K875R	Ubiquitination
		915	239	2	p.Y915C/H	Phosphorylation
		940	263	1	p.T940A	Phosphorylation
EPHA3	EphA2_TM	561		1	p.Y561F	Phosphorylation
		602		1	p.Y602C	Phosphorylation
EPHA5	Pkinase_Tyr	676	45	1	p.T676A	Phosphorylation
		710	76	1	p.K710N	Ubiquitination
		822	189	1	p.S822F	Phosphorylation
		856	217	2	p.T856I	Phosphorylation
EPHA7	EphA2_TM	601		1	p.K601T	Ubiquitination
	Pkinase_Tyr	666	74	1	p.T666N	Phosphorylation
EPHB1	EphA2_TM	575		1	p.Y575F	Phosphorylation
		582		1	p.Y582F	Phosphorylation
		588		1	p.S588F	Phosphorylation
EPHB2	EphA2_TM	575		1	p.S575L	Phosphorylation
		578		1	p.T578M	Phosphorylation
		585		1	p.T585I	Phosphorylation
		602		1	p.Y602F	Phosphorylation

Gene	Domain	Position	PKA	#Mutation	MutationAA	PTM
ERBB2	Pkinase_Tyr	724	48	1	p.K724N	Ubiquitination
		733	57	5	p.T733I	Phosphorylation
FGFR2	Pkinase_Tyr	587		1	p.S587C	Phosphorylation
		616	157	1	p.Y616D	Phosphorylation
		733	267	1	p.Y733H	Phosphorylation
FLT3	Pkinase_Tyr	614	48	1	p.K614N	Ubiquitination
		759		1	p.S759L	Phosphorylation
		772		2	p.K772N	Ubiquitination
		842		6	p.Y842C/H	Phosphorylation
HCK	Pkinase_Tyr	311	98	2	p.T311I/S	Phosphorylation
		412		2	p.T412K/M	Phosphorylation
		442	224	1	p.S442F	Phosphorylation
KIT	Pkinase_Tyr	721		1	p.Y721H	Phosphorylation
		735		2	p.K735E/M	Ubiquitination
		801	176	2	p.T801I	Phosphorylation
		821	194	2	p.S821Y/F	Phosphorylation
		823		59	p.Y823D/C/N/H	Phosphorylation
LCK	Pkinase_Tyr	246	45	1	p.K246N	Ubiquitination
		269	69	1	p.K269E	Ubiquitination
		281	85	1	p.S281F	Phosphorylation
MAP2K1	Pkinase	72	48	1	p.S72G	Phosphorylation
		212	189	1	p.S212N	Phosphorylation
		231	207	1	p.S231L	Phosphorylation
MAP2K3	Pkinase	222	198	15	p.T222M	Phosphorylation
		230	205	1	p.Y230H	Phosphorylation
		243	214	1	p.K243T	Ubiquitination
PAK2	PBD	112		2	p.T112I	Phosphorylation
		128		10	p.K128R	Acetylation
		130		1	p.Y130N	Phosphorylation
PRKACA	Pkinase	49	49	1	p.T49I	Phosphorylation
		54	54	1	p.S54F	Phosphorylation
		280	280	1	p.K280E	Acetylation
						Ubiquitination
PRKCB	Pkinase	352	54	3	p.S352N/G	Phosphorylation
		498	196	2	p.T498I/S	Phosphorylation
		504	202	1	p.T504N	Phosphorylation
		515	213	1	p.Y515F	Phosphorylation
	Pkinase_C	632		4	p.K632Q	Methylation
		661		6	p.S661F/C	Phosphorylation
PRKCQ	Pkinase_C	685		4	p.S685I	Phosphorylation
		695		1	p.S695F	Phosphorylation
RET	Pkinase_Tyr	791	108	3	p.Y791N/F	Phosphorylation
		891	184	10	p.S891A/L	Phosphorylation
		904		2	p.S904Y/L	Phosphorylation
TTN	PPAK	10296		3	p.P10296A/R/C	Phosphorylation
		10297		1	p.A10297D	Phosphorylation
		10313		1	p.T10313N	Phosphorylation
	fn3	21842		1	p.S21842F	Phosphorylation
ZAP70	Pkinase_Tyr	492		1	p.Y492C	Phosphorylation
		500	201	1	p.K500R	Ubiquitination
		506	206	1	p.Y506H	Phosphorylation

Table S1. Mutation-PTM overlapping sites in enriched domains. PKA: PKA position; MutationAA: mutation amino acid (wild-type, position, and mutant type).

a

```
PREFIX prokino: <...>
PREFIX pro: <...>
PREFIX nextprot: <...>
PREFIX mgi: <...>

SELECT ?UniProtID ?Mutation_Count ?Reaction_Count ...
WHERE
{
    #Backbone: UniProt IDs
    {
        #Protein Kinases Defined By ProKinO
        #Service provider: ProKinO
        SERVICE <http://vulcan.cs.uga.edu/sparql>  {...}
    }
    #Variables from ProKinO
    OPTIONAL
    {
        #Service provider: ProKinO
        SERVICE <http://vulcan.cs.uga.edu/sparql>
        {
            #Count ?Mutation
            {
                SELECT ?UniProtID COUNT(?Mutation) AS ?Mutation_Count
                WHERE
                {
                    SELECT DISTINCT ?UniProtID ?Mutation
                    WHERE
                    {
                        ?Gene prokino:hasDbXref ?UniProtID .
                        ?Gene prokino:hasMutation ?Mutation .
                    }
                }
                GROUP BY ?UniProtID
            }
            OPTIONAL {...} #Count ?Reaction
            OPTIONAL {...} #Count ?Complex
            OPTIONAL {...} #Count ?Pathway
            OPTIONAL {...} #Count ?PubMed_Human
        }
    }
    #Variables from PRO
    OPTIONAL
    {
        #Service provider: PRO
        SERVICE <http://sparql.proconsortium.org/virtuoso/sparql>
        {
            {...} #Count ?Homologs
            OPTIONAL {...} #Count ?Modification
        }
    }
    #Variables from neXtProt
    OPTIONAL
    {
        #Service provider: neXtProt
        SERVICE <https://sparql.nexptrot.org> {...}
    }
    #Variables from MGI
    OPTIONAL
    {
        #Service provider: MGI (via Bio2RDF)
        SERVICE <http://bio2rdf.org/sparql> {...}
    }
}
```

b

```
PREFIX prokino: <...>
PREFIX pro: <...>
PREFIX nextprot: <...>
PREFIX mgi: <...>

SELECT ?UniProtID ?PKA ?Motif ?Pathway ...
WHERE
{
    #Backbone: UniProt IDs
    {
        #Protein Kinases Defined By ProKinO
        #Service provider: ProKinO
        SERVICE <http://vulcan.cs.uga.edu/sparql>  {...}
    }
    #Variables from ProKinO
    OPTIONAL
    {
        #Service provider: ProKinO
        SERVICE <http://vulcan.cs.uga.edu/sparql>
        {
            SELECT ?UniProtID ?PKA ?Motif ?Pathway ...
            WHERE
            {
                ?Gene prokino:hasDbXref ?UniProtID .
                ?Gene prokino:hasMutation ?Mutation .
                ?Mutation prokino:hasPKAStartLocation ?PKA .
                ?Gene prokino:participatesIn ?Pathway .
                ...
            }
        }
    }
    #Variables from PRO
    OPTIONAL
    {
        #Service provider: PRO
        SERVICE <http://sparql.proconsortium.org/virtuoso/sparql>
        {
            SELECT ?Proteoform ?Category ?Modification ...
            WHERE {...}
        }
    }
    #Variables from neXtProt
    OPTIONAL
    {
        #Service provider: neXtProt
        SERVICE <https://sparql.nexptrot.org>
        {
            SELECT ?Component ?Function ?Process ...
            WHERE {...}
        }
    }
    #Variables from MGI
    OPTIONAL
    {
        #Service provider: MGI (via Bio2RDF)
        SERVICE <http://bio2rdf.org/sparql>
        {
            SELECT ?Expression ?Component ?Function ...
            WHERE {...}
        }
    }
}
```

Figure S1. Pseudocodes of high-level and low-level federated queries. (a) Pseudocode of high-level federated query. (b) Pseudocode of low-level federated query. All executable queries are available at https://github.com/esbg/SPARQL.

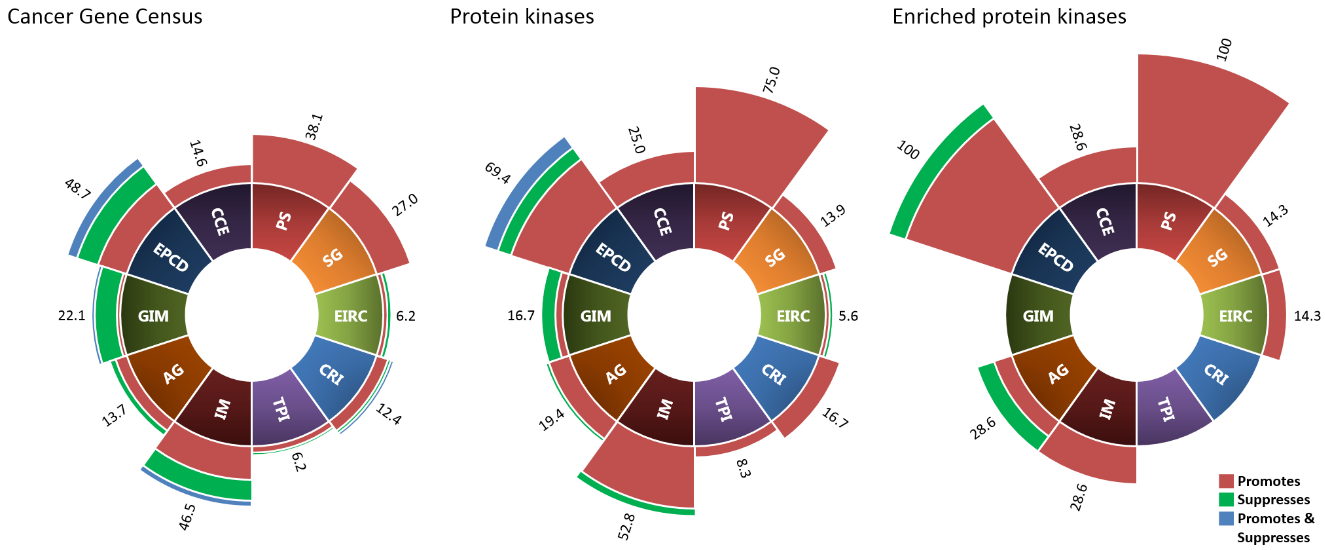

Figure S2. Hallmark distribution. The radial stacked bar charts show the percentage of each hallmark (the number of genes associated with the hallmark, including all roles– promotes (red), suppresses (green), or both (blue), divided by the total number of genes in the following different gene sets: all the genes in Cancer Gene Census (left), protein kinases (center), and protein kinases with enriched PTM/mutation domains (right)). PS: proliferative signalling; SG: suppression of growth; EIRC: escaping immunic response to cancer; CRI: cell replicative immortality; TPI: tumour promoting inflammation; IM: invasion and metastasis; AG: angiogenesis; GIM: genome instability and mutations; EPCD: escaping programmed cell death; CCE: change of cellular energetics. Data were collected from COSMIC Cancer Gene Census[5].

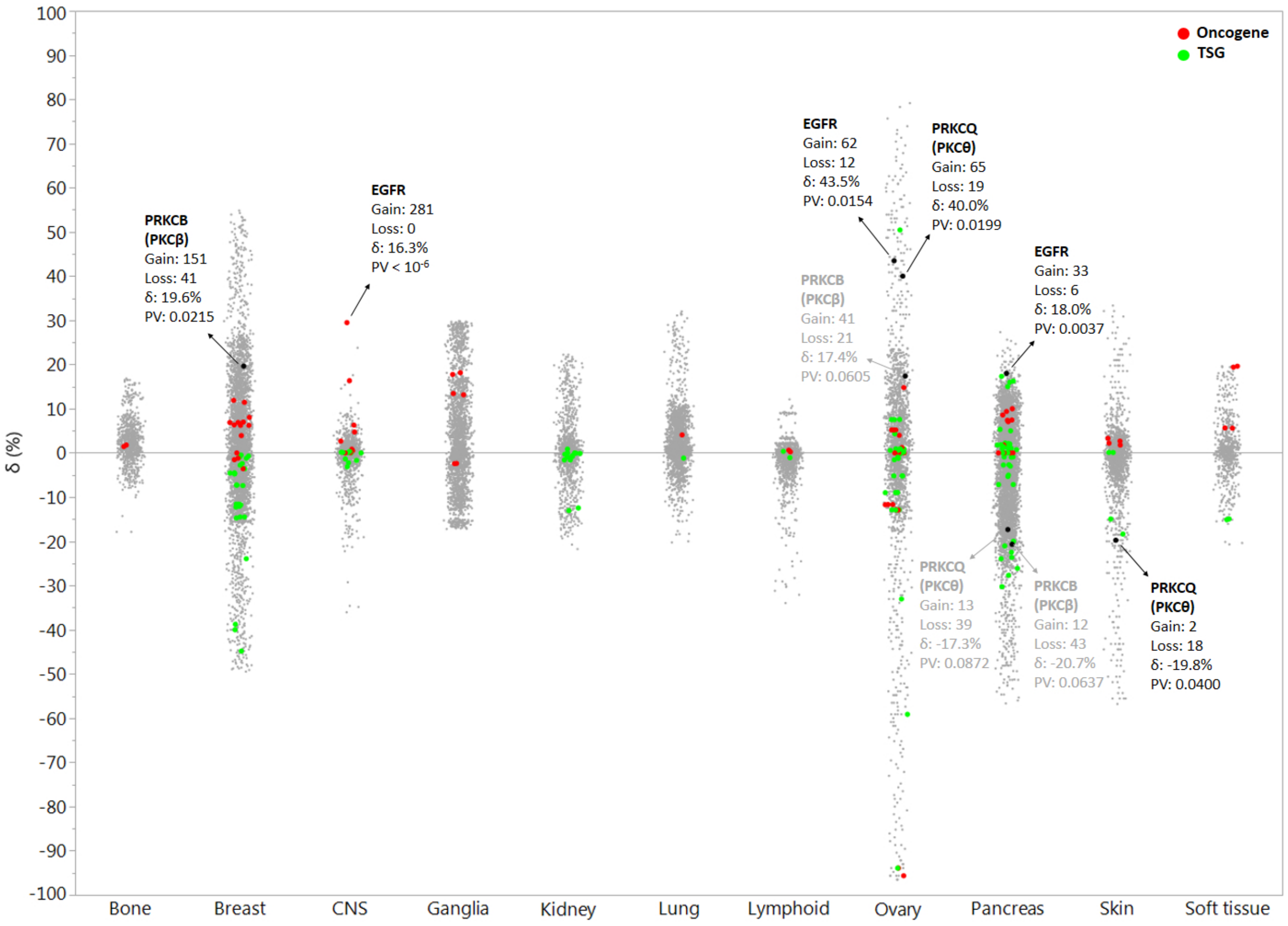

Figure S3. Copy number variation analysis. Gray dots: non-protein kinase genes; red dots: known oncogenes of the corresponding cancer subtype; green dots: known tumour suppressor genes (TSGs) of the corresponding cancer subtype. Significant amplification/deletion of the three case study genes are labelled by black text, while the statistics of the three case study genes near the boundary of significant level are labelled by gray text. CNS: central nervous system; Ganglia: autonomic ganglia; Lymphoid: haematopoietic and lymphoid tissue.
